# Summarising salient information on historical controls: A structured assessment of validity and comparability across studies

**DOI:** 10.1177/1740774520944855

**Published:** 2020-09-21

**Authors:** Anthony Hatswell, Nick Freemantle, Gianluca Baio, Emmanuel Lesaffre, Joost van Rosmalen

**Affiliations:** 1 Department of Statistical Science, University College London, London, UK; 2 Delta Hat Limited, Nottingham, UK; 3 Institute of Clinical Trials and Methodology, University College London, London, UK; 4 L-Biostat, KU Leuven, Leuven, Belgium; 5 Department of Biostatistics, Erasmus MC, Rotterdam, The Netherlands

**Keywords:** Historical control, power prior, propensity scoring, matching-adjusted indirect comparison

## Abstract

**Background:**

While placebo-controlled randomised controlled trials remain the standard way to evaluate drugs for efficacy, historical data are used extensively across the development cycle. This ranges from supplementing contemporary data to increase the power of trials to cross-trial comparisons in estimating comparative efficacy. In many cases, these approaches are performed without in-depth review of the context of data, which may lead to bias and incorrect conclusions.

**Methods:**

We discuss the original ‘Pocock’ criteria for the use of historical data and how the use of historical data has evolved over time. Based on these factors and personal experience, we created a series of questions that may be asked of historical data, prior to their use. Based on the answers to these questions, various statistical approaches are recommended. The strategy is illustrated with a case study in colorectal cancer.

**Results:**

A number of areas need to be considered with historical data, which we split into three categories: outcome measurement, study/patient characteristics (including setting and inclusion/exclusion criteria), and disease process/intervention effects. Each of these areas may introduce issues if not appropriately handled, while some may preclude the use of historical data entirely. We present a tool (in the form of a table) for highlighting any such issues. Application of the tool to a colorectal cancer data set demonstrates under what conditions historical data could be used and what the limitations of such an analysis would be.

**Conclusion:**

Historical data can be a powerful tool to augment or compare with contemporary trial data, though caution is required. We present some of the issues that may be considered when involving historical data and what (if any) statistical approaches may account for differences between studies. We recommend that, where historical data are to be used in analyses, potential differences between studies are addressed explicitly.

## Background

Clinical investigations of novel interventions are usually performed as randomised controlled trials (RCTs) against placebo or a relevant comparator. Although RCTs in which all patients are randomised to either the intervention or control treatment are the standard approach for comparing efficacy, there is interest in making use of historical data to reduce the need for contemporary controls. The historical data can, in principle, increase the power of tests for treatment efficacy and improve precision of the estimates. In some cases (e.g. in rare/orphan diseases), the use of historical data is necessary to obtain a sufficiently powered analysis, due to the limited number of patients available for randomisation. In principle, both intervention and control patients of previous studies can be combined with data of the current study. Previous studies typically do not feature exactly the same intervention and control treatment, and especially the intervention may change between trials. Therefore, the focus lies on combining historical control patients with patients randomised to the control in the current study. The combination of historical and randomised controls to supplement the analysis of contemporary or future clinical trials was popularised by Pocock.^1^ The main concern with the use of historical data is the possibility that the historical studies differ in characteristics with the current study, due to, for example, improvement in supportive care over time, differences in patient selection, and between-centre differences. Indeed, previous work has demonstrated substantial bias when comparing the outcomes of comparisons made using observational data to RCTs conducted in the same population.^2–7^ This finding was also quantified in a simulation study.^8^

**Figure 1.**
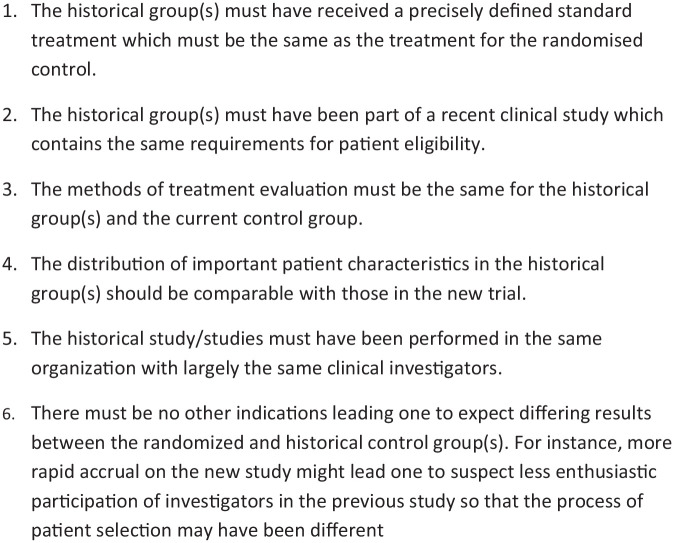
Acceptability conditions proposed by Pocock regarding the use of a historical control group.

To overcome the issues discussed above, Pocock suggested criteria that should be met for the historical data to be deemed ‘acceptable’. These criteria are presented in Figure 1. Provided the criteria are met, the suggestion is that the historical data may be combined with contemporary randomised controls, thereby reducing the number of patients required in the control arm. Since the use of historical controls was originally proposed, their application has proliferated beyond the pooling of placebo data from sequential studies to include areas, such as sample size calculations, synthesis of published historical data, and comparative efficacy analyses for treatments, which are granted a marketing authorisation on the basis of uncontrolled studies.^9,10^ Additional relevant criteria for the acceptability of historical data are drift, exchangeability, and conditional exchangeability.^11^ Drift can be defined as a bias (difference in underlying model parameters) between the historical data and current data, which can arise due to general improvements in supportive care over time. Exchangeability implies that the historical studies and the current study constitute a random sample from a population of studies, whereas conditional exchangeability implies that the studies constitute a random sample from a population of studies only after accounting for differences in patient characteristics. Conditional exchangeability applies more generally than exchangeability. Under these criteria, meta-analytic approaches for combining the historical and current controls may be applied. The use of historical data has proven to be a useful tool for studies on orphan diseases,^12,13^ whereas it remains controversial in the primary analysis of a phase III RCT.

Historical controls may originate from a previous study of the same group/institution, from the literature, or from other sources (for example, patient registries). However, such data only occasionally meet the criteria set out originally by Pocock in an exact manner, which specify that the historical studies must have been conducted by the same investigators, have similar patient characteristics, and must have been done in roughly the same time period. Since such cross-study comparisons are of considerable interest, one could make use of techniques, such as propensity score matching,^14^ among others,^15^ which allow some of these barriers to be addressed. Depending on the reason for the use of historical controls and the purpose of the analysis, some of the Pocock criteria could be relaxed with the use of appropriate methodology without introducing bias. Likewise where multiple studies are available, there may be a desire to use all studies, but with different weightings.

Given the developments in statistical methodology, trial design, and medical research practice since the 1976 paper of Pocock, the objective of this article is to describe an updated tool for assessing the similarity between historical and contemporary data. With this aim in mind, we develop a set of questions which may help identify areas that differ between studies (whether in design, patients, or setting). We also discuss potentially appropriate statistical methods by which historical data may still be utilised in case data sets are not perfectly aligned. The application of the resulting tool is illustrated with an example of cetuximab compared to standard care in colorectal cancer. Our hope is that by highlighting the relevant issues (and signposting methodologies) the tool enables analysts to better understand the issues with historical data and justify the choice of methodology used for analysis.

## Methods

We aim to identify the relevant different aspects between historical and current studies that should be taken into account when historical data are considered to be included in the analysis. Pocock’s original criteria serve as the starting point for the development of the tool. In addition, we considered the statistical assumptions implicit in the use of historical data, and which differences between studies could result in violations of those assumptions. These statistical considerations are described in Supplemental Appendix 1, together with the resulting insights used to augment the proposed tool.

In order to ascertain whether historical data set(s) may be sufficiently similar for use alongside current data (for instance, with some form of combining), the process by which outcomes were obtained should be understood. This means there is a need to compare the treatments given and the circumstances of the studies–for example, patient characteristics. We therefore divide the relevant issues into three areas (outcome measurement, study and patient characteristics, and disease process and intervention effects) and discuss each in turn. These issues are illustrated statistically in Supplemental Appendix 1 and described in non-technical language below. These areas are subsequently used to derive questions that can help identify the relevant differences between studies.

### Outcome measurement

One of the changes that can occur over time is the way outcomes are measured. For example, as technology has advanced, tests have become more sensitive, and definitions have evolved – for example, the widely used Common Terminology Criteria for Adverse Events (CTCAE) are now at version 4.0. There have also been changes in the types of outcomes used in studies over time, for instance, moving from response rates to median survival, and then subsequently beyond to endpoints such as restricted mean survival time.^16^ Any differences in how outcomes are measured/defined (or in the type of outcome) should therefore be understood and accounted for – for example, by reanalysis of the contemporary trial or mapping between endpoints – even if outcomes are named similarly, for example, partial response, the definition may have changed over time. If the differences between studies cannot be bridged, the historical data may need to be discarded.

### Study and patient characteristics

In order to combine the historical data with current data, the inputs to the process by which outcomes are generated, that is, the interaction of the disease process and the mechanism of action of any interventions, must remain similar. In practice, this implies that the study inclusion criteria and the individual patient characteristics should be assessed for similarity between the historical studies and the current study. If these are not fully aligned, for example, if the historical patients and the current patients exhibit different characteristics, statistical methods accounting for this imbalance across studies may be required (which are likely to, correctly, increase the uncertainty around estimates).

If there is little overlap in patient characteristics or if there are structural differences in inclusion criteria, the historical studies and current study may be incompatible, such that no statistical adjustment method would be able to overcome the unquantifiable bias. An example here would be if some but not all studies required patients to first complete a ‘wash-out’ period from their previous treatment. This implies that only patients who survive to the beginning of the study (a form of immortal time bias)^17^ are included in the results.

### Disease process and intervention effects

Furthermore, the way in which outcomes are achieved must be similar across studies. However, differences may arise for several reasons. For example, changes in supportive care over time may improve outcomes, even if the intervention remains unchanged. Alternatively, interventions may change over time as their usage is refined, or protocols in different centres may prescribe a different usage. For example, the use of stem cell transplantation has been continually refined over time, with improvements in both short-term and long-term mortality.^18^

It is also possible that the impact of the disease changes over time – either as screening improves resulting in earlier diagnosis (and patients with a more favourable outcome) or as a new treatment is introduced at early stage disease, meaning patients who do progress have much more severe disease subtypes. It is also possible that the disease itself changes in the case of pathogens, which may evolve over time or become resistant to treatments (as seen with influenza, bacterial infection, and HIV).

### Application to illustrative example: cetuximab in metastatic colorectal cancer

**Figure 2.**
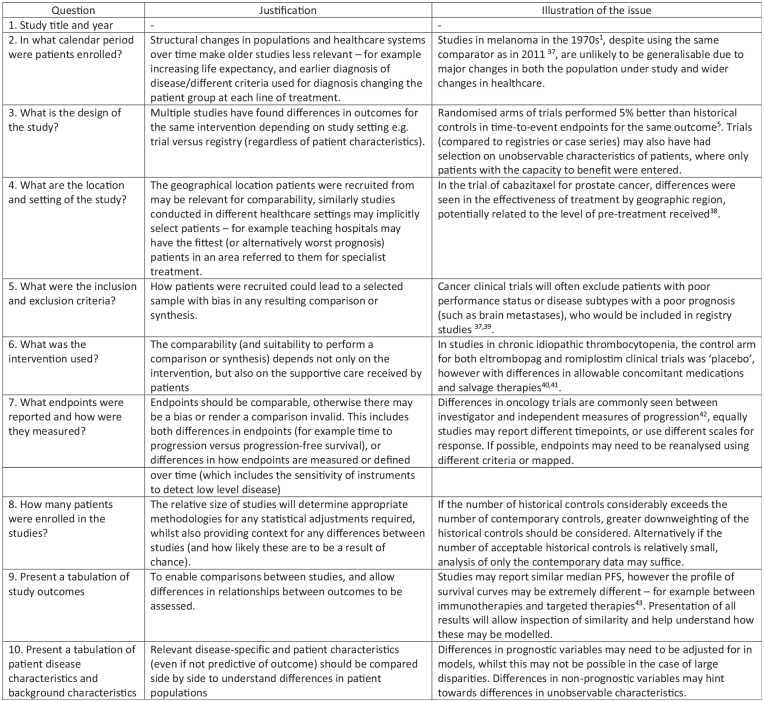
Motivating example: Cetuximab in metastatic colorectal cancer.

To illustrate and evaluate the proposed framework, it was applied to a motivating example of a historical controlled study conducted by Annemans et al.^19^ of cetuximab in metastatic colorectal cancer (mCRC) (Figure 2). Cetuximab was licenced on the basis of the BOND study (see Cunningham et al.^20^), an RCT of 329 patients which compared cetuximab plus irinotecan versus cetuximab monotherapy in 11 centres across Europe. This study design addressed the benefit of combination therapy, which was shown to provide a higher response rate than monotherapy (22.9% versus 10.8%), but did not provide an estimate of the efficacy of cetuximab versus standard care. To make such a comparison for the benefit of reimbursement agencies, Annemans et al.^19^ conducted a retrospective review of patient notes to identify untreated patients from the largest centres in BOND. This investigation was conducted in the three largest centres, enrolling 66 patients who received standard care in the same centres, but outside the period the cetuximab trial was ongoing. The tool was therefore used to evaluate the comparison of these two studies.

## Results

**Figure 3.**
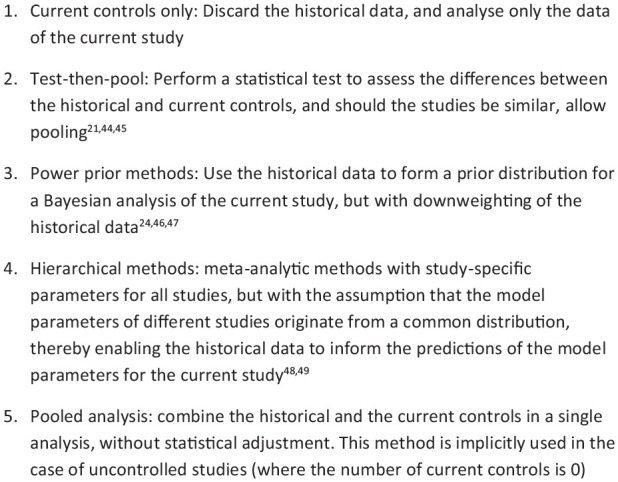
Questions and justifications regarding important items for historical controls.

Using Pocock’s criteria as a starting point, we updated and more elaborately described the issues that are relevant for incorporating historical data, which yielded the proposed tool for assessing historical data that is presented in Figure 3. In this figure, each issue is highlighted with a reference to the literature illustrating the issue.

The areas of importance are the patient characteristics, precise intervention used (including supportive care), outcome measurement, and patient selection to the study. The statistical/methodological rationale for the question as well as an example of how studies may be non-comparable and the impact on any comparison(s) are given alongside each question. The tool in Figure 3 may be used to identify areas where historical controls differ from contemporary data, and as a result whether statistical adjustments may be required, or where there are such important differences that only narrative comparisons are warranted.

The items in the tool do have a large degree of overlap with the criteria proposed by Pocock; however, they are different in nature. Rather than proposing a set of dichotomous criteria that must all be met to allow for pooling or synthesis of the studies, our proposed tool instead seeks to present relevant data and study design aspects and to identify and quantify differences, giving a more nuanced picture. This is in line with the aim of comparing between studies and allowing a judgement to be made of the appropriate next steps. The main additions we make to the original criteria pertain to the study design and the patient selection, which in the original Pocock criteria were taken to be placebo arms from the same centre. The tool also asks for the study results to be presented for comparison, as these may indicate differences between studies (if the same intervention is used in both arms) or be required for the use of statistical methods such as ‘test then pool’ as a next step.^21^

### Illustrative example: cetuximab in mCRC

The study by Annemans et al. appears to be of high quality in attempting to identify similar patients to those from the clinical trials, from the same centres and date range – although imperfect, it is a pragmatic attempt to estimate the outcomes of standard care without availability of RCT or network of trials.

Using our proposed tool, it is apparent that, although the largest centres from the Cunningham et al.’s study were used as the source of control data, there may be differences between these studies and the other centres enrolled – due to their size or their location (Belgium, France, and Italy) being unrepresentative. To present a valid comparison between combination therapy and standard care, a useful next step would be to inspect the subsample of the Cunningham et al.’s study using only the centres enrolled in the Annemans et al.’s study. The patient characteristics for the Annemans et al.’s study are not given in the publication even at the aggregate level – to be confident in the use of these data as a historical control, the distribution of patient characteristics should be shown.

Provided there is good overlap between the study characteristics and patient characteristics within the studies, the use of the Annemans et al.’s data as a historical control would seem appropriate, provided it is used with suitable statistical techniques (which may involve either matching or weighting methods). To perform any statistical adjustments in this example, access would be required to the patient-level data from both studies. The main limitation of such a comparison is also highlighted through the use of our proposed table, which clearly identifies that only overall survival (OS) is available in the Annemans et al.’s data set. Comparisons on the primary endpoint of the Cunningham et al.’s study (response rate) or time to progression are not possible without the use of further assumptions.

### Appropriate statistical methods

A variety of methods has been proposed to account for differences in observed patient characteristics between historical and contemporary data. These methods include the aforementioned use of not only propensity scores but also meta-regression,^22^ matching-adjusted indirect comparisons,^23^ simulated treatment comparisons,^24^ and other regression-based techniques^25^– each of which have varying data requirements.

**Figure 4.**
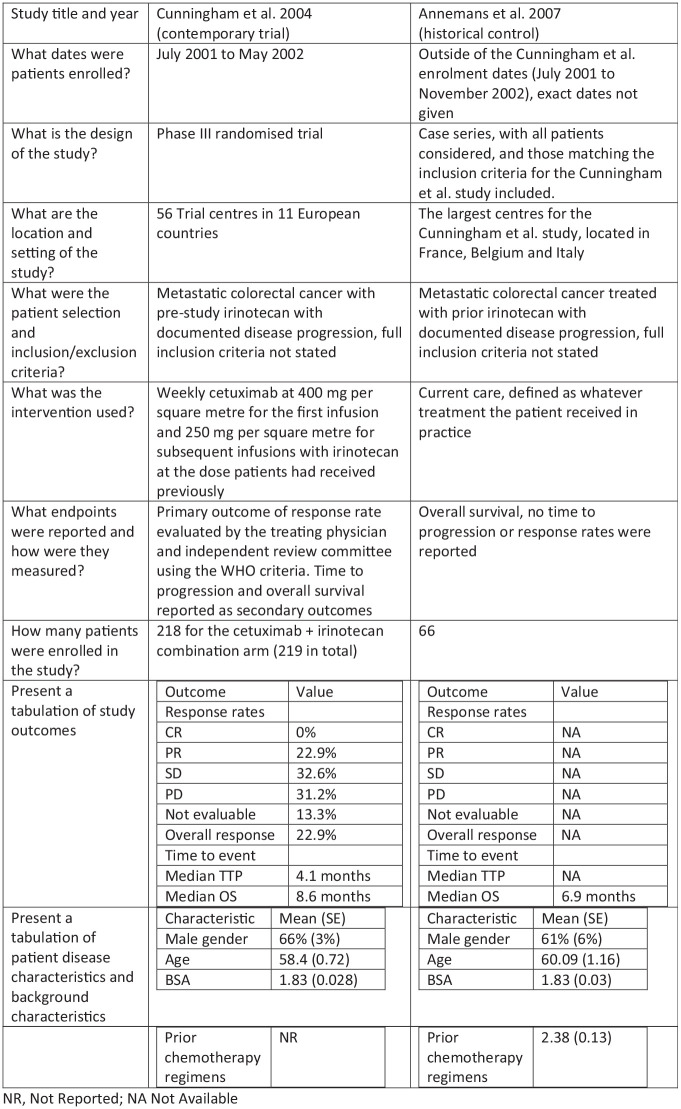
Recognised statistical methods for the use of historical data.

If there is a desire to perform a combined analysis of historical and contemporary data, an appropriate statistical method or methodology to adjust for study-specific effects and other potentially unobservable differences between studies is required; see Wadsworth et al.^26^ for a systematic review. A selection of techniques that perform adjustments for between-study differences is presented in Figure 4, with the ordering of techniques based on how stringent the required assumptions regarding comparability are. These techniques range from simply discarding the historical data to ‘naïve’ pooling of historical and contemporary evidence. The former of these methods is appropriate if there are irreconcilable differences between historical and current controls, while the latter is acceptable only if the historical and current controls originate from essentially the same study – a criterion that seldom applies.

The remaining three highlighted methods (test then pool, power priors, and hierarchical methods) can be used in intermediate situations, where there are no insurmountable differences in outcome measures, patient characteristics, and disease process/intervention effect, but some adjustment is still necessary. Most implementations of these methods perform ‘dynamic borrowing’, that is, the information in the historical controls is given a lower weight in the analysis depending on the size of the observed differences between historical and contemporary controls – the more similar the data, the higher weight assigned to the historical data. Recent simulation studies have shown that the three intermediate methods (test-then-pool, power prior, and meta-analytic methods) may suitably account for between-study differences and may also be used to control the type I error rate. In particular, the meta-analytic methods appear to lead to the largest reduction in bias and ability to control the type I error rate.^27^ New variants and adaptations of these methods are frequently proposed such as a test-then-pool approach based on equivalence tests,^28^ extensions of the power prior to data with multiple historical studies, and combinations of these methods.^29^ However, none of these methods are guaranteed to control the type I error rate at the nominal 5% level.^30^ While these techniques are aimed at accounting for between-study heterogeneity (which is unobserved), in the case of observed differences, for example, patient characteristics, these methods may need to be augmented either with adjustment techniques or by including the relevant patient characteristics as covariates in the statistical model.

The ‘naïve’ pooling of studies is the final method in the list, which implies that no kind of statistical adjustment is made for differences between historical and current controls. We believe that there are applications where pooling data may be appropriate, however, only if the strictest criteria for comparability are met, and between-study differences can virtually be ruled out. Potential examples of such applications are the example given in the 1976 paper (sequential studies in a single centre, if appropriate) and some studies used for US Food and Drug Administration approvals. At times, two RCTs are conducted in parallel with essentially the same protocol and time frame, but in different centres – should these circumstances be met, patients may be allocated by the instrument of geography into one study or the other. Even in such cases (effective allocation by time or geography) between-study differences should be investigated and naïve pooling should be used with extreme caution.

## Discussion

The questions identified in our tool lead to a summary of relevant information of the historical controls. From this information, we can identify where data are suitable for pooling (i.e. rarely), where they could be used with statistical adjustment, or alternatively where the review may highlight that an informative comparison between studies is not possible. The questions included in the table represent key areas where studies may differ, collated based on Pocock’s original criteria, statistical theory, and the experience of the authors. The novelty of this tool is that it provides a framework for systematically comparing a historical study to a current study, using both the observed data and the study designs, while leaving necessary room for debate. Except for the work of Pocock, few authors have discussed or proposed clear criteria for choosing historical data. See the work of Lim et al.^31,32^ for a broader review of general principles to consider when selecting and incorporating historical control data.

Because a single major difference can render a comparison between studies inappropriate (in line with the general experience from quality scores for clinical studies), we do not suggest to use the tool to score trials in an objective and quantitative manner. However, we hope that in answering the questions presented, a strong basis for a decision on the comparability of studies may be achieved. When compared with Pocock’s original criteria, the questions ask for details of the studies, instead of asking if they are the same. This is in keeping with the different objective, of understanding the similarity of studies for further analysis – we then highlight a variety of techniques that may aid the analyst in conducting such pooling. While there is an unavoidable need for judgement of similarity, and when different methods would be appropriate, we would hope that this at least makes such decisions explicit, rather than implicit.

The proposed criteria do not yield an unequivocal decision of whether the historical data are to be considered ‘acceptable’. However, the criteria should enable researchers to make an assessment of whether there is a risk of drift (systematic bias in the historical data compared to the current data) and whether the assumption of exchangeability or conditional exchangeability is reasonable. Exchangeability is an assumption that is relatively difficult to demonstrate and communicate, whereas drift (bias) seems more straightforward. Nevertheless, we believe that both drift and exchangeability are useful criteria for assessing historical data and choosing an appropriate analysis method. Previous simulation work showed that some dynamic borrowing methods are relatively robust as long as the historical data are (conditionally) exchangeable, whereas even a small systematic bias in the historical data threatened the performance of these methods and led to inflated type I error rates.^27^ Drift due to improvements in care over time is also an important concern in practice. In a meta-analysis by Snyders et al.,^33^ who looked at 63 trials of docetaxel in lung cancer (enrolling over 10,000 patients), it was found that outcomes improved each year by a mean of 0.3% in objective response rate (ORR), 0.5% for progression-free survival (PFS), and 0.9% in OS. These changes over time imply that historical data that are more than say 10 years old for docetaxel in this indication should be avoided. The rate of drift in other diseases will differ, but the potential for change is something the analyst should be aware of.

The application of the tool is illustrated by our motivating example, where it highlights the differences between studies and leads naturally to the next steps required for comparisons to be drawn. In the majority of cases, these next steps will consist of further statistical analysis which may be as simple as trimming data sets to ensure entry criteria are similar or may involve more complex modelling as mapping between outcomes and weighting of patients. Where multiple studies are available, more complex techniques may be needed such as those highlighted in Figure 4. The tool may also highlight that the differences between studies are too great, so that some or all historical studies should be omitted from the analysis.

The main advantage of the proposed tool is that in a side-by-side comparison, differences between studies are highlighted, and their importance can be discussed as opposed to simply referred to as a ‘historical control’. The tool can then be presented as a single table or figure in a journal article or evidence dossier for submission to regulatory bodies or payers. Its use would also not represent additional burden, as should historical data be used, it is reasonable to expect the detail of the comparison to be provided (if anything, this burden should reduce).

The main limitation of the proposed tool is that it is not possible to know whether there exists a bias even in trials that appear superficially similar. This is particularly the case in the case of uncontrolled studies – in such instances, it is not possible to compare control arms to assess between-study variation, which has been highlighted in the literature as an area of concern^34^ and for future research.^25^ Despite this limitation, uncontrolled studies with carefully selected historical controls should be preferable to uncontrolled studies that compare observed outcomes to an assumed outcome so that sampling variability in the historical data is ignored, or, even worse, when the response rate under the null hypothesis lacks a clear data-based justification.^35^ Similarly, there may be other biases present; for instance, a sponsor may only commission a Phase 3 study following promising results in Phase 2 – a finding which has been seen with medical interventions, in general.^36^ Beyond these limitations, we would also note that the particular tools that should be used for statistical adjustments are not specified and do rely on judgement as there is no easily quantifiable decision rule or flowchart to choose the type of statistical technique.

While the use of historical controls does not represent the highest level of data on the ‘evidence pyramid’, in many circumstances, such as rare cancers, orphan diseases, and some Class 3 medical devices, their use is necessary. In other cases, appropriate use of historical data may be seen to be more ethical by reducing the need to expose patients to control treatments and reducing the cost of trials. The tool we present may help improve the quality and appropriateness of such historical comparisons in a variety of settings. With appropriate use of historical data (collected at great financial and human cost), the quality of decision-making by clinicians, regulators, and payers could improve, ultimately leading to better patient outcomes.

## Supplemental Material

Appendix_r0-3_clean – Supplemental material for Summarising salient informationon historical controls: A structured assessment of validity and comparability across studies Click here for additional data file. Supplemental material, Appendix_r0-3_clean for Summarising salient informationon historical controls: A structured assessment of validity and comparability across studies by Anthony Hatswell, Nick Freemantle, Gianluca Baio, Emmanuel Lesaffre and Joost van Rosmalen in Clinical Trials
