# Supplementary material for: Summarising salient information on historical controls: A structured assessment of validity and comparability across studies
Source: Clin Trials. 2020 Sep 21;17(6):607–16. doi: 10.1177/1740774520944855 (PMC7649932; doi:10.1177/1740774520944855)
Supplement: Appendix_r0-3_clean – Supplemental material for Summarising salient informationon historical controls: A structured assessment of validity and comparability across studies [file Appendix_r0-3_clean.pdf]

Appendix: Statistical considerations

Terms and definitions

We assume that there are H historical studies and 1 current (contemporary) study, with indices $h = 1, \ldots, H$ denoting the historical studies, and index $h = H + 1$ denoting the current study. Typically each study consists of a control arm and an investigational arm, but for the historical studies, only the control arms are included in the analysis. The control arm of each study comprises data of n_h patients, with outcome y_{hi} for patient i in study h. The outcome $y_{hi}$of individual patients ($i, i = 1, \ldots, n_i$) in study h is distributed as

$$y_{hi} \sim f(\beta_h, x_{hi1}, \ldots, x_{hip}),$$

with $x_{hi1}, \ldots, x_{hip}$ describing the individual patient characteristics for patient *i* in study *h*, parameter $\beta_h$describing the impact of these characteristics on the outcome, and $f()$ describing the distribution of the data. This general specification allows for outcomes with different measurement levels, such as continuous and dichotomous outcomes as well as survival data.

Assumptions for the parameters of the historical data

Several assumptions for the parameters are possible, but all assumptions made should follow from an evaluation of acceptability criteria. The strongest assumption, required for complete pooling of the data, is that patients are exchangeable between studies. In statistical terms this means that β_h must be identical for all studies, implying that the distribution of the outcome after adjustment for the exposures and patient characteristics is the same for each study, and that the samples will only differ on the basis of sampling variability. This assumption is however unlikely to be met; even if two studies are conducted in the same population and setting, a study-specific or centre-specific effect typically cannot be ruled out.

A somewhat more lenient assumption is exchangeability at the study level, which means that all studies (including the contemporary study) can be seen as a random sample from a population of studies. In formal terms, exchangeability at the study level implies that the study-specific parameter β_h follows a normal distribution with overall mean μ_β and variance σ_β^2, so that $\beta_h \sim N(\mu_\beta, \sigma_\beta^2)$. Exchangeability would for instance be violated in the following circumstances:

1. If some of the historical studies are more similar in design to each other than to the remaining studies
2. If there is a natural ordering in the trials (e.g. year in which study was conducted) that is relevant for the distribution of the outcome.
3. If the current study is different from all historical studies in some aspect of its design.

Typically, the historical studies have been conducted over a period of time, which means that the exchangeability assumption may not easily be satisfied in practice. The potential effects of changes in the distribution of the outcome over time should thus be carefully assessed.
If the statistical model includes other model parameters that are not assumed to vary across studies, for example to model the effects of patient characteristics, exchangeability of the study populations would only be assumed conditional on the patient characteristics. Conditional exchangeability may thus still apply in situations where the distribution of patient characteristics differs between studies, provided that appropriate adjustments are made for the effects of patient characteristics. This means that the relevant patient characteristics should be included in the statistical model, that the model should be correctly specified (e.g. including interactions and nonlinear effects), and measured in the same way in each trial. Substantial differences in the distribution of patient characteristics

would require out-of-sample predictions which may be unreliable. Adjustment for patient characteristics is of course only possible if individual-level data of the historical studies are available.

In some cases the assumption of (conditional) exchangeability at the study level should not be considered reasonable. The acceptability of the historical data could then be determined by assessing what level of drift is likely given the study designs and study characteristics, where drift is defined as the difference in model parameters between the current (β_{H+1}) and the historical studies ($\beta_h, h = 1 \ldots, H$). If the study designs and study characteristics, as evaluated using our table, show that the level of drift can be expected to be small, the historical data may still prove useful, even if the studies are not exchangeable. However previous simulation results have shown that most borrowing methods are not robust to moderate or larger levels of drift, which would lead to inflation of the type I error rates [1]. Note that the level of drift is not observable; researchers would need to be able to rule out the possibility of a large level of drift a priori.

Implications for the tool

- Over time due to changes in the population there may be changes in $f()$, or in x_{hij} which may or may not be observable.
- The study design may affect the inclusion criteria, even if not explicitly stated, leading to a selection bias in x_{hij}.
- Different prevalence or selection on observables and unobservable patient characteristics may result in differences in x_{hij} between studies, e.g. so-called confounding by indication. Equally differences between populations may affect the values of coefficients (β_h).
- Differences in the intervention (incorporated via a treatment effect in β_h) or the incorrect intervention for the decision problem should be identified.
- A systematic bias in y_{hi} will emerge if endpoints are not measured in the same way at the same time.
- The uncertainty in estimates of β_h will be affected by the number of patients, whilst the statistical techniques for performing adjustment will also vary.
- y_{hi} between studies should be consistently reported, and have similar relationships to any required surrogate outcomes (if relevant).

The exchangeability assumption is in principle sufficient for several methods for combining historical and current controls, such as the meta-analytic approach proposed by Neuenschwander[2]. However, the amount of heterogeneity (the variance σ_β^2) should preferably be limited, otherwise the historical data will be of little help for predicting the parameter β_{H+1}in the contemporary trial. In some cases, it may be necessary to allow multiple parameters to vary across trials, for example when the effects of patient characteristics differ between trials. A multivariate version of the meta-analytic approach could then be needed, which may be difficult to estimate in situations with limited data. In practice it is convenient to only assume exchangeability for the most important parameters, such as the intercept measuring baseline risk in logistic regression models, and assume other parameters to be equal across studies.

In situations where the exchangeability assumption is not satisfied for the current study, for example due to a time trend in the distribution of the outcome, there will be a bias in the model parameters for the current controls, i.e. $\beta_{H+1} \sim N(\mu_\beta^*, \sigma_\beta^2)$ for the current study and $\beta_h \sim N(\mu_\beta, \sigma_\beta^2)$ for the historical studies, with $\mu_\beta^* \neq \mu_\beta$. There is, by definition, no statistical adjustment available for such an unknown bias, and the power for detecting such biases using the observed data may be limited. Historical data may only be used if there is a reasonable expectation, based on comparison of study designs characteristics of the patients, that the potential for such fundamental differences is small or absent.
